# Reticular Erythematous Mucinosis Mimicking a Vascular Dermatosis: A Clinicopathologic Diagnostic Challenge

**DOI:** 10.7759/cureus.114636

**Published:** 2026-08-17

**Authors:** Bouchra Idrissi-Rhenimi, Lina Mouline, Laetitia Goudetsidis, Geraldine Titeca

**Affiliations:** 1 Dermatology, Université Mohammed V de Rabat, Rabat, MAR; 2 Dermatology, Clinique Notre-Dame de Grâce, Gosselies, BEL

**Keywords:** clinicopathological correlation, dermoscopy, differential diagnosis, pseudo-vascular eruption, reticular erythematous mucinosis

## Abstract

Reticular erythematous mucinosis (REM) is a rare primary cutaneous mucinosis characterized by dermal mucin deposition and a chronic, indolent clinical course. Because of its nonspecific clinical presentation, REM may mimic a broad spectrum of inflammatory, connective tissue, and vascular dermatoses, frequently resulting in delayed diagnosis and inappropriate management.

We report the case of a 57-year-old man with no significant past medical history who presented with a four-year history of a slowly progressive, occasionally pruritic eruption involving the upper back. The lesions gradually enlarged before stabilizing and failed to respond to multiple courses of topical corticosteroids. Clinical examination revealed grouped erythematous macules and papules coalescing into a reticulated erythematous plaque with a pseudo-vascular appearance. Dermoscopic examination performed with a polarized DermLite DL5 (3Gen Inc., San Juan Capistrano, CA, USA) demonstrated a diffuse pink background with fine irregular telangiectasias and multiple whitish structureless areas. Routine laboratory investigations, including antinuclear antibody testing, were unremarkable. Histopathological examination revealed a superficial and deep perivascular and periappendageal lymphohistiocytic infiltrate, while Alcian blue staining demonstrated dermal mucin deposition. Immunohistochemical analysis showed a predominantly T-cell infiltrate with scattered B lymphocytes, rare CD30-positive cells, and a few plasma cells, supporting a reactive inflammatory process. Integration of the clinical, dermoscopic, histopathological, and immunohistochemical findings established the diagnosis of REM. As the eruption was minimally symptomatic and clinically stable, the patient declined treatment, and no disease progression was observed during follow-up.

This case expands the clinical spectrum of REM by illustrating an uncommon pseudo-vascular presentation in a male patient. It underscores the pivotal role of clinicodermoscopic-pathologic correlation in establishing the diagnosis of atypical presentations and avoiding unnecessary investigations, inappropriate treatments, and diagnostic delay.

## Introduction

Reticular erythematous mucinosis (REM) is a rare primary cutaneous mucinosis characterized histopathologically by dermal mucin deposition associated with a superficial and deep perivascular and periappendageal lymphocytic infiltrate. It predominantly affects middle-aged women and typically presents as slowly progressive erythematous papules or plaques with a reticulated configuration involving the midline chest or upper back [1-4]. Owing to its rarity and nonspecific clinical presentation, REM remains underrecognized and may be misdiagnosed as inflammatory, connective tissue, or vascular dermatoses, leading to delayed diagnosis and inappropriate management [1,3,4].

The pathogenesis of REM remains incompletely understood. Proposed mechanisms include immune dysregulation, ultraviolet radiation, hormonal influences, and autoimmune associations, although none has been consistently confirmed [1,3,4]. Histopathological examination combined with Alcian blue staining remains the diagnostic cornerstone, as routine hematoxylin-eosin sections alone may not sufficiently demonstrate dermal mucin deposition [1,5].

Dermoscopy is a non-invasive imaging technique that allows in vivo visualization of subsurface cutaneous structures not readily visible to the naked eye. Despite increasing recognition of REM, published descriptions of its dermoscopic features remain limited, and their diagnostic significance remains incompletely characterized. In this report, we present a case of REM with an atypical pseudo-vascular presentation and discuss the diagnostic value of clinicodermoscopic-pathologic correlation in light of the current literature.

## Case presentation

A 57-year-old man with no significant past medical history presented to our Dermatology Department with a four-year history of a slowly progressive eruption involving the upper back. The lesions had gradually increased in extent during the first months before remaining clinically stable. The patient reported occasional pruritus but denied pain, photosensitivity, systemic symptoms, or seasonal exacerbations. Multiple courses of topical corticosteroids prescribed by his primary care physician had failed to improve the lesions. As the eruption was minimally symptomatic, the consultation was mainly prompted by his wife's concern regarding its persistent appearance.

Dermatological examination revealed multiple erythematous macules and papules coalescing into reticulated plaques distributed predominantly over the upper back, with a pseudo-vascular appearance characterized by fine telangiectatic erythema (Figure 1). No scales, atrophy, induration, ulceration, or mucosal involvement were observed. The remainder of the cutaneous examination was unremarkable.

**Figure 1:**
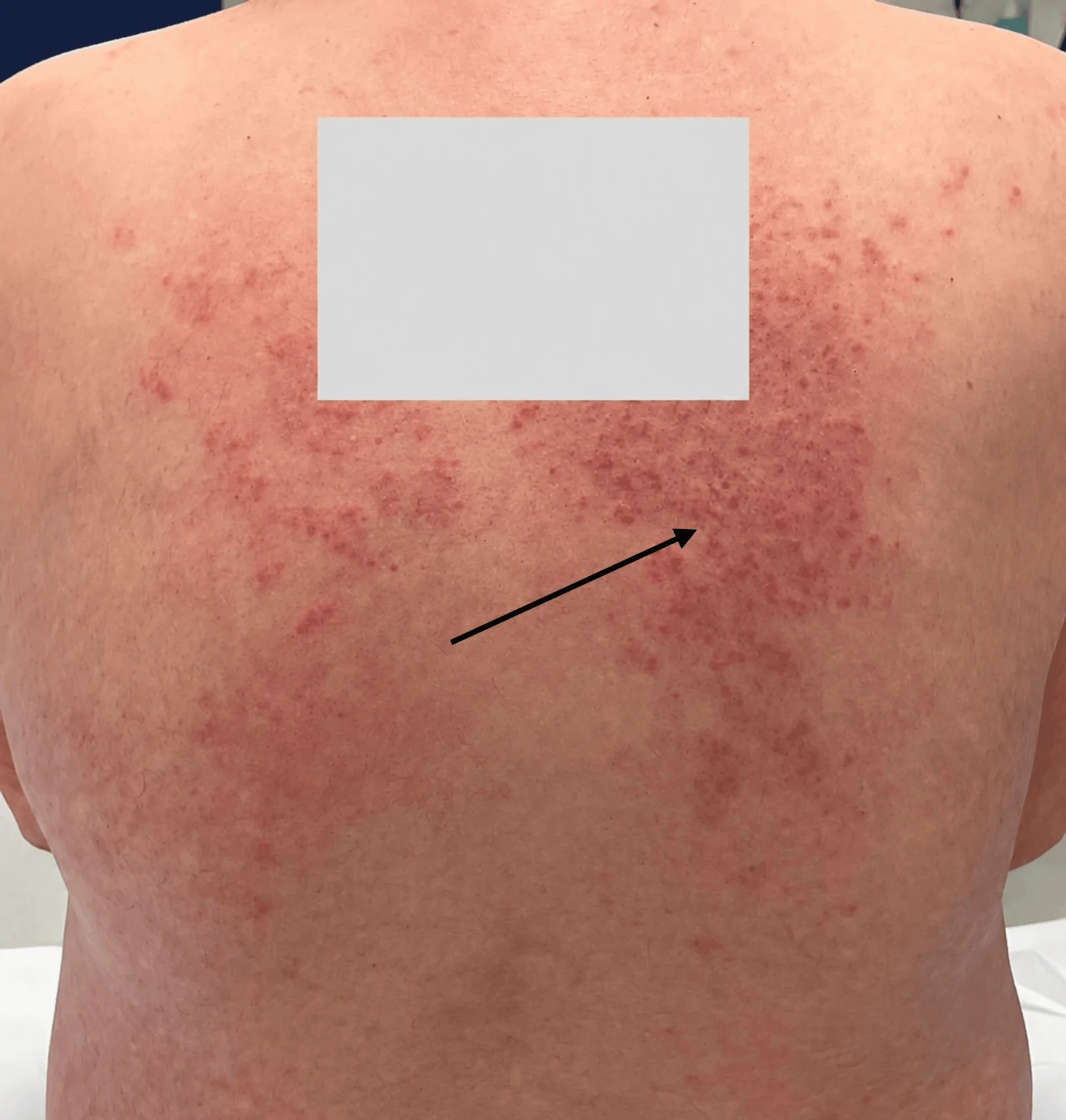
Clinical presentation of reticulated erythematous plaques on the upper back Clinical presentation of reticulated erythematous plaques on the upper back. Clinical photograph showing multiple erythematous macules and papules coalescing into reticulated plaques with a pseudo-vascular appearance over the upper back. Arrow highlight representative areas of the reticulated pattern.

Dermoscopic examination was performed using a polarized handheld dermoscope (DermLite DL5, 3Gen Inc., San Juan Capistrano, CA, USA), revealing a diffuse pink background traversed by fine irregular linear telangiectatic vessels associated with multiple whitish structureless areas (Figure 2). Although these findings were not specific, they suggested an inflammatory rather than a primary vascular process.

**Figure 2:**
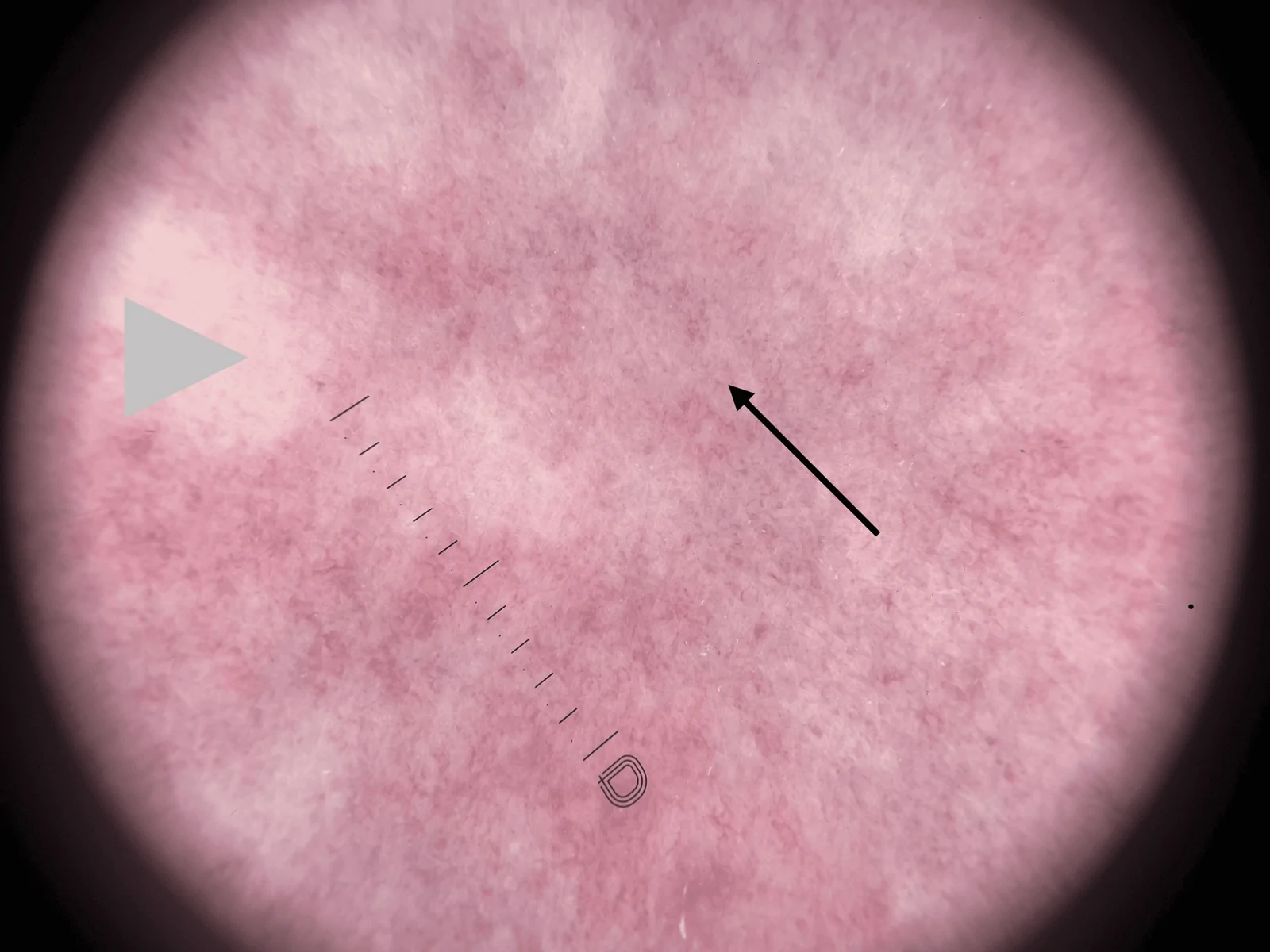
Dermoscopic features of reticulated mucinous erythematous lesions Dermoscopic features of reticulated mucinous erythematous lesions. Polarized dermoscopy showing a diffuse pink erythematous background with fine irregular telangiectatic vessels and multiple whitish structureless areas. Arrows highlight representative telangiectatic vessels and whitish structureless areas.

Routine laboratory investigations, including complete blood count, erythrocyte sedimentation rate (ESR), C-reactive protein (CRP), liver and renal function tests, were within normal limits. Antinuclear antibody testing was negative.

Given the chronic course, atypical clinical presentation, and absence of response to topical corticosteroids, a punch biopsy was performed. Histopathological examination revealed a superficial and deep predominantly perivascular and perisudoral lymphohistiocytic infiltrate without significant epidermal changes. Alcian blue staining demonstrated dermal mucin deposition, consistent with cutaneous mucinosis. Immunohistochemical analysis supported a reactive inflammatory infiltrate and excluded a primary cutaneous lymphoid proliferation. Based on the clinical, dermoscopic, histopathological, and immunohistochemical findings, a diagnosis of REM was established.

The diagnosis and therapeutic options, including antimalarial treatment, were discussed with the patient. Because the lesions had remained stable for several years and caused only minimal symptoms, he declined treatment. Clinical reassessment at follow-up showed no evidence of lesion progression.

## Discussion

REM is a rare primary cutaneous mucinosis of unknown etiology, characterized by dermal mucin deposition associated with a superficial and deep perivascular and periappendageal lymphocytic infiltrate. It predominantly affects middle-aged women and usually presents as erythematous reticulated plaques involving the midline of the chest or upper back. Although several pathogenic mechanisms have been proposed, including ultraviolet radiation, hormonal influences, immune dysregulation, and chronic inflammation, the exact pathogenesis remains uncertain [1-4].

Our patient illustrates several atypical aspects. First, the patient was male, whereas REM demonstrates a marked female predominance in the largest published series [3,4]. Second, the lesions evolved slowly over approximately four years before stabilizing and remained almost completely asymptomatic except for occasional mild pruritus. Finally, the clinical morphology consisted of confluent erythematous macules and papules producing a striking pseudo-vascular appearance that initially suggested disorders such as unilateral nevoid telangiectasia, acquired vascular malformations, angioma serpiginosum, telangiectatic mastocytosis, or an early cutaneous lymphoproliferative disorder. Similar atypical presentations have only occasionally been reported, emphasizing the broad clinical spectrum of REM [6-8].

Dermoscopic features of REM have been only rarely described, and their diagnostic significance remains incompletely characterized [9,10]. In our patient, polarized dermoscopy demonstrated a diffuse pink background with fine irregular linear telangiectatic vessels associated with multiple whitish structureless areas. Although these findings are not specific, they are consistent with the clinicopathological findings and support an inflammatory rather than a primary vascular process. Additional reports correlating dermoscopic and histopathological findings are needed to better define the dermoscopic spectrum of REM and facilitate earlier recognition of this uncommon disorder [9,10].

Histopathological examination remains the cornerstone of diagnosis, particularly in clinically atypical cases. In our patient, hematoxylin-eosin staining demonstrated a superficial and deep predominantly perivascular and periappendageal lymphohistiocytic infiltrate extending into the deep dermis. Alcian blue staining confirmed dermal mucin deposition, establishing the diagnosis of cutaneous mucinosis. Immunohistochemical analysis demonstrated a predominantly CD3-positive T-cell infiltrate with scattered CD20-positive B lymphocytes, preserved CD4/CD8 balance, rare CD30-positive cells, and a few CD138-positive plasma cells, supporting a reactive inflammatory infiltrate and arguing against a primary cutaneous lymphoid proliferation. These findings, interpreted in conjunction with the clinical presentation, established the diagnosis of REM while excluding its principal histopathological mimickers [5].

The differential diagnosis of REM includes lupus erythematosus tumidus, Jessner lymphocytic infiltration, polymorphous light eruption, pseudolymphoma, telangiectatic disorders, and early cutaneous lymphoma [1,4,5,11]. In our patient, the absence of photosensitivity, systemic symptoms, and antinuclear antibodies, together with normal inflammatory markers, made connective tissue disease less likely. The pseudo-vascular clinical appearance initially raised the possibility of a primary telangiectatic disorder; however, histopathology showed a predominantly perivascular and periappendageal lymphohistiocytic infiltrate rather than a primary vascular proliferation. Finally, the presence of dermal mucin on Alcian blue staining, together with the predominantly reactive T-cell infiltrate on immunohistochemistry, supported REM and argued against a primary cutaneous lymphoproliferative disorder. Because these entities may exhibit overlapping clinical and histopathological features, clinicopathological correlation together with special stains demonstrating dermal mucin remain essential for establishing the correct diagnosis [5].

Hydroxychloroquine is considered the first-line treatment for REM and has demonstrated favorable outcomes in most published cases [1,3,4,6]. In contrast, topical corticosteroids generally provide limited or inconsistent benefit, as illustrated by our patient, who experienced no clinical improvement despite repeated treatment. Because the lesions had remained stable for several years and caused only minimal symptoms, the patient declined systemic therapy after receiving detailed counseling regarding the benign nature of the disease.

As a single-case report, our observations have limited generalizability and should be interpreted accordingly. In addition, the patient declined systemic treatment because of the minimal symptoms and long-term stability of the lesions; therefore, clinical follow-up was pursued, with no evidence of progression during follow-up.

## Conclusions

REM should be considered in the differential diagnosis of chronic reticulated erythematous eruptions, even in male patients and in the absence of photosensitivity or other classic clinical features. This case highlights the potential for REM to mimic vascular and lymphoproliferative disorders, making clinicopathological correlation essential. Histopathological examination with Alcian blue staining remains the key diagnostic tool, while dermoscopy may provide useful supportive clues, although its features are not yet well established. Additional clinicopathologic studies and dermoscopic descriptions are needed to better define the diagnostic spectrum of REM.
